# Isolated rectal bleeding in neonates: a scoping review

**DOI:** 10.1016/j.jped.2026.101594

**Published:** 2026-08-12

**Authors:** Alexandra Markevich, Júlia G. Botelho, Luana L. Morikawa, Maria Ângela Bellomo Brandão, Elizete A. Lomazi

**Affiliations:** a Universidade Estadual de Campinas (UNICAMP), Faculdade de Ciências Médicas (FCM), Department of Pediatrics, Campinas, SP, Brazil; b Universidade Nove de Julho, São Paulo, SP, Brazil; c Universidade Estadual de Campinas (UNICAMP), Faculdade de Ciências Médicas (FCM), Division of Pediatric Gastroenterology, Campinas, SP, Brazil

**Keywords:** Newborn, Hemorrhage, Rectal bleeding, Proctocolitis

## Abstract

**Objective:**

To review the etiological factors that cause isolated rectal bleeding in otherwise asymptomatic neonates.

**Data source:**

A scoping review was conducted following PRISMA-adapted guidelines. The databases consulted included PubMed, PubMed Central, BVS, Scopus, Embase, Web of Science, Cochrane, PROQUEST, and EBSCOHOST, with no language or publication period restrictions. The authors reviewed studies of any methodological design, including neonates up to 28 days of life with visible blood in the stool, with no symptoms of disease.

**Summary of the findings:**

A total of 1,287 articles were identified, and 749 duplicates were removed. After screening the title and abstract, 139 studies were selected for full-text review, of which 56 were included. The data included 1,154 neonates. The diagnoses, in decreasing frequency order, were isolated rectal bleeding (IRB), cow’s milk protein allergy (CMPA), swallowing maternal blood, hemorrhagic colitis, intestinal infections, and nodular lymphoid hyperplasia. Around 22% of patients diagnosed with CMPA underwent oral food challenge to confirm the diagnosis.

**Conclusions:**

Most of the reviewed articles presented case reports or observational case series. The main cause of rectal bleeding in otherwise asymptomatic neonates was the descriptive diagnosis of isolated rectal bleeding, which evolved benignly and spontaneously. The second most frequent cause was CMPA, though most cases were not confirmed by an oral food challenge. IRB in neonates has been managed without standardized protocols for investigation or treatment.

## Introduction

Isolated rectal bleeding (IRB) is characterized as visible blood in the stool of infants up to 28 days old that occurs without associated symptoms.

IRB is a clinical manifestation that, although generally benign and self-limiting, often causes significant concern among healthcare professionals and family members, particularly due to its potential association with serious neonatal conditions, such as necrotizing enterocolitis (NEC) and intestinal infections. At the same time, the absence of clinical or radiological signs in these cases makes their interpretation challenging and often subject to divergent management approaches.

The clinical significance of this condition stems from a few studies showing an increase in cases in recent decades [1,2]. This increase is associated with diagnostic uncertainty and practical management issues.

A review of this topic is warranted by the scarcity of consolidated data regarding its prevalence, etiologies, and clinical course, as well as by the heterogeneity of diagnostic criteria and therapeutic approaches described in the literature.

Furthermore, a review can help better distinguish cases truly associated with significant pathologies from those with a benign course, promoting a more rational, evidence-based approach and reducing unnecessary interventions.

## Methods

A scoping review of the literature was conducted based on the “Preferred Reporting Items for Systematic Reviews and Meta-Analyses” (PRISMA) method, adapted for scoping reviews(3). The research question was “What are the etiological factors that cause isolated rectal bleeding in otherwise asymptomatic neonates?” The population included neonates from birth up to 28 days old who had visible blood in their stools and were otherwise asymptomatic.

The databases included were PubMed, PubMed Central, BVS, Scopus, Embase, Web of Science, Cochrane, PROQUEST, and EBSCOHOST. A search strategy was developed for each database (Appendix I). The search strategy included the descriptors and their respective synonyms: “infant, newborn,” “hemorrhage,” “gastrointestinal hemorrhage,” “rectum,” “rectal bleeding.”

No restrictions were applied regarding publication date or language, and studies were not excluded based on design.

Review registration: Open Science Framework; 2022. https://osf.io/sdn6z/.

Duplicate results were removed using the Rayyan website [4]. Initial screening was performed independently by two reviewers, assessing titles and abstracts. Full texts were then retrieved and evaluated for eligibility. In cases of initial disagreement between reviewers, consensus was reached through discussion. Additional references were assessed through forward search (articles cited by the selected studies) and backward search (articles citing the selected studies).

### Inclusion criteria

Studies of any methodological design addressing neonates up to 28 days old with visible blood in the stool and no clinical symptoms. Patients diagnosed with cow’s milk protein allergy by resolution of bleeding following removal of the heterologous protein from their diet were not excluded and were classified as such.

### Exclusion criteria

Studies with incomplete clinical data regarding age that could not be evaluated after contacting the authors.

As this was a scoping review using data already available in the literature, without direct use of patient data, the study did not require ethics committee approval.

### Data extraction

After full-text reading, data were extracted regarding authorship, year of publication, country, study design, number of children included, final diagnosis, colonoscopy findings (if performed), and notes considered relevant to the review.

## Results

**Figure 1:**
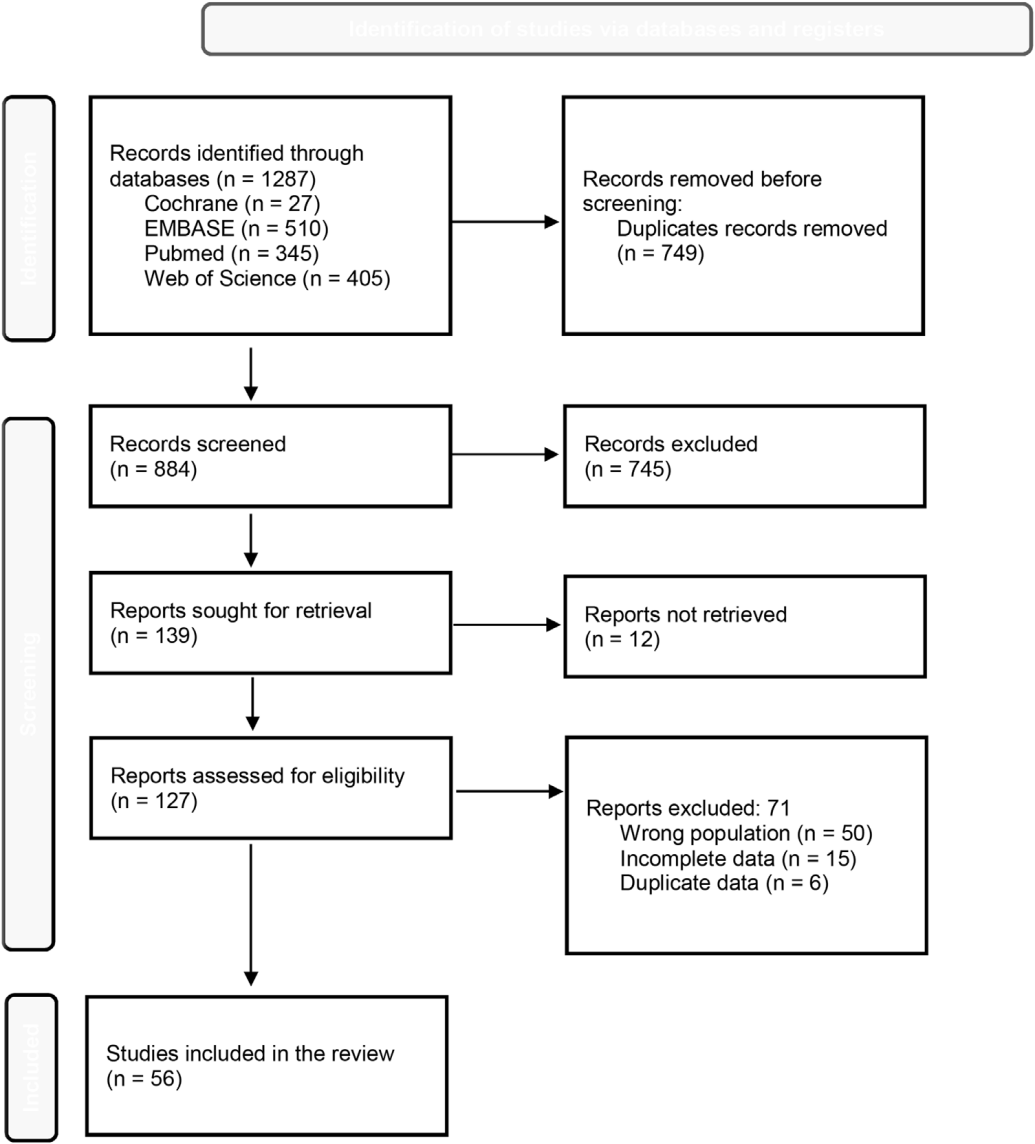
Flowchart for study selection.

The flowchart for the selection and inclusion of studies is shown in Figure 1.

Of the 56 selected studies, 21 were case series, three were case-control studies, and 32 were case reports. The details of the studies are provided in Appendix II [1,5., 6., 7., 8., 9., 10., 11., 12., 13., 14., 15., 16., 17., 18., 19., 20., 21., 22., 23., 24., 25., 26., 27., 28., 29., 30., 31., 32., 33., 34., 35., 36., 37., 38., 39., 40., 41., 42., 43., 44., 45., 46., 47., 48., 49., 50., 51., 52., 53., 54., 55., 56., 57., 58., 59.]. The publication period of the included studies ranged from 1979 to 2025, and as for the origin, 27 were from Europe, 17 were from Asia, 11 were from North America, and one was from Oceania.

The etiologies and relative frequencies of isolated rectal bleeding cases are presented in Table 1 [1,3,6., 7., 8., 9., 10., 11., 12., 13., 14., 15., 16., 17., 18., 19., 20., 21., 22., 23., 24., 25., 26., 27., 28., 29., 30., 31., 32., 33., 34., 35., 36., 37., 38., 39., 40., 41., 42., 43., 44., 45., 46., 47., 48., 49., 50., 51., 52., 53., 54., 55., 56., 57., 58., 59.].

**Table 1:** Etiologies attributed to isolated rectal bleeding.

Etiology	Number of cases	References
**Isolated rectal bleeding**	**603**	(1,3,6-8,10-12,24,26,27)
**CMPA**	**294**	
Positive OFC	31	(9,10,20-22,24,32,34,37,39,49)
OFC not performed	110	(7,9,12,17,18,23,25,28,30,33,35,38,45)]
No information on OFC	153	(26)
**Swallowed maternal blood**	**51**	(26,56)
**Infections**	**44**	
Viral infections	33	(23,26)
*Campylobacter* infection	7	(31,48,50,53)
Amebiasis	4	(52)
**Colitis and enteritis**	**127**	
Hemorrhagic colitis	46	(7)
Neonatal transient colitis	30	(20,22,27)
Allergic colitis	28	(11,14,42)
Ecchymotic colitis	17	(14)
Transient eosinophilic colitis	4	(47,57-59)
Eosinophilic gastroenteritis	1	(44)
FPIES	1	(29)
**Gastrointestinal malformations**	4*	
Meckel's diverticulum	2	(40,54)
Colonic angiodysplasia	2	(13,41)
Colonic duplication	1	(40)
**Others**		
Anal fissure	22	(11,23,25,26)
Nodular lymphoid hyperplasia	4	(15,16,19)
Intramural hematoma	1	(43)
Maternal medication	1	(55)
Drug (sildenafil)	1	(51)
Vaginal bleeding	1	(12)
Gastric ulcer	1	(46)

CMPA, Cow’s milk protein allergy; OFC, oral food challenge. FPIES, Food protein-induced enterocolitis syndrome.

⁎ One patient presented with Meckel’s diverticulum and colonic duplication simultaneously (40).

Among the 141 patients reported with a final diagnosis of cow’s milk protein allergy, only 31 (21.9%) underwent oral food challenge confirming the diagnosis [9,10,20., 21., 22.,^24^,^27^,^32^,^34^,^37^,^39^,^49^]. The study conducted by Lin et al., published in 2022, reported 153 patients with cow’s milk protein allergy as the cause of rectal bleeding, but explicitly stated that it did not distinguish between those who underwent oral food challenge and those who did not. For this reason, these patients were excluded from this calculation [26].

## Discussion

Most of the publications included in this review were observational studies that described case reports or case series. The case reports primarily identified the following entities as a diagnosis of IRB: cow’s milk allergy, transient eosinophilic colitis, and certain vascular malformations. The case series were primarily retrospective, and the cases were managed without a defined protocol. Laboratory investigations were conducted in a non-systematic manner, as is common in retrospective observational studies. After excluding NEC, many patients were treated with fasting or suppression of cow's milk protein. For most patients, the outcome was spontaneous cessation of bleeding without any conclusive etiological investigations being performed. While these studies are valuable for generating hypotheses and providing descriptive characterizations, they offer limited evidence for clinical decision-making.

A total of eight case series involving 603 patients were given the descriptive diagnosis of "isolated rectal bleeding" when no recognized nosological entity was identified through investigations and the neonates remained asymptomatic. This descriptive diagnosis was the most common cause of IRB reported in studies included in this review, accounting for half of all cases. The second most common diagnosis was cow’s milk protein allergy. This diagnosis was confirmed either by eliminating heterologous protein from the diet followed by bleeding cessation, or by performing an oral food challenge. This confirmatory test was performed on approximately 22% of patients. The next most frequent diagnoses were infectious colitis and enteritis, followed by anal fissure. These diagnoses accounted for 10.5%, 3.6%, and 1.8%, respectively, of the patients in the case series.

Only two of the studies reported IRB incidence [1,2]. A single-center study [1] reported on a cohort of 49,705 term or near-term neonates born between 1996 and 2001. The authors reported that 183 infants (0.37%) experienced rectal bleeding, with no significant differences observed between full-term and near-term neonates. More recently, Cordero et al. conducted a study including preterm infants admitted to neonatal intensive care units and reported incidence rates of 0.6% between 2000 and 2005, which increased significantly to 2.1% between 2005 and 2010 (p < 0.01) [2].

Patients who met the definition of isolated rectal bleeding had a benign and self-limiting clinical course. In a retrospective case-control study conducted in Israel, Maayan-Metzger et al. [1] compared 147 neonates with IRB to 147 healthy controls. They found that IRB was more prevalent among formula-fed infants than among breastfed infants. Clinical courses evolved benignly in all cases after one to three days without feeding, and in 43% of cases antibiotics were used for five days with resolution of bleeding and no requirement for further diagnostic or therapeutic intervention. Absence of breastfeeding was associated with bleeding, conferring a more than fourfold increase in risk among formula-fed infants.

The diagnosis of isolated rectal bleeding in neonates was not determined using a single method. A rectosigmoidoscopy with a rectal mucosal biopsy is considered the most effective way to determine the cause of bright red blood in the stool [60]. Based on this gastroenterological paradigm, let us review the evidence the authors have identified in this review.

Due to the potential risks of an endoscopic procedure for necrotizing enterocolitis, this procedure has not been systematically performed on neonates with isolated rectal bleeding [8,10].

The authors identified 21 case series and three case-control studies; 12 of them performed an endoscopic evaluation, primarily a rectosigmoidoscopy for investigating IRB. Nine studies included small samples, ranging from one to six neonates per study. Endoscopic findings varied among patients within each sample and differed across studies. The most frequently reported findings were nodular lymphoid hyperplasia on macro and eosinophilic infiltrate in mucosal biopsies. These histological findings were used to confirm allergic proctocolitis and CMPA was considered the final diagnosis.

It is worth noting the findings of a group of researchers who presented their endoscopic results for neonates who bled before their first feeding. They coined the term "transient neonatal eosinophilic colitis" to describe the condition, which differs from CMPA, because the bleeding occurred before exposure to heterologous protein. According to the report, the two “patients were in a clinically stable condition, and their abdomens were soft. The results of their blood analyses, abdominal radiographs, and stool cultures were normal, but they had gross eosinophilia and resolved spontaneously” [19,20]. It seems to be a descriptive diagnosis that has not yet been explained on a pathophysiological basis as CMPA.

Three studies systematically performed rectosigmoidoscopy with biopsy on neonates with IRB. Taxman et al. [11] reported their findings from 51 neonates. Dupont et al. [10] evaluated 34 neonates and Canioni et al. [14] examined 18 infants aged from six to 38 days old.

Taxman et al. [11] included a heterogeneous group, for whom cow's milk allergy was a plausible and confirmed diagnosis. All neonates (4–28 days old) were fed cow’s milk; 46 had endoscopic evidence of inflammation ranging from mild to severe; and 24 presented with eosinophilic infiltrates in the mucosa; 10 neonates progressed to necrotizing enterocolitis. In all patients, bleeding ceased with fasting. The authors concluded that there were no macroscopic differences between patients who developed NEC and others, likely because the NEC cases were mild, and that cow’s milk protein allergy is a common cause of isolated rectal bleeding in neonates; however, the diagnostic method for allergic proctitis was not specified in the study; oral food challenge has not been cited.

Dupont et al. [10] reported the results of rectosigmoidoscopy in 34 neonates with IRB in whom intestinal infection, anal fissure, hemorrhagic disease of the newborn, and necrotizing enterocolitis had been ruled out, according to the authors. All patients had a history of clinical morbidity, prematurity, small for gestational age, a high frequency of blood transfusions, exchange transfusion, respiratory distress, and umbilical catheterization. All 34 newborns had a spontaneous and favorable outcome. The endoscopic findings were characterized by the presence of ecchymotic lesions on a normal or congested mucosa, absence of eosinophilic infiltrates, and frequent hemorrhages in the rectum and/or left colon — a condition for which the authors coined the term “ecchymotic colitis.”

Canioni et al. [14] presented the results of a histological evaluation of samples collected via rectosigmoidoscopy from 18 infants with isolated rectal bleeding, in whom ecchymotic colitis was identified. Seventeen patients had a benign course; one patient progressed to necrotizing enterocolitis. As in the series by Dupont et al. [10], the endoscopic lesions were all similar: “ecchymotic patches, or longitudinally oriented stripes on normal or congested mucosa, separated by intervals of normal mucosa.” Histology revealed mild to moderate inflammation with a predominance of polymorphonuclear or lymphomonocytic cells, few eosinophils, an absence of ulcers or erosions, and focal superficial hemorrhages. Four patients presented with *pneumatosis coli* of the mucosa and submucosa. The authors explained their findings, supported by histology, by suggesting that these patients had a mild or early form of necrotizing enterocolitis that resolved spontaneously without specific treatment.

Taken together, the studies with rectosigmoidoscopy in larger case series showed histopathological findings with eosinophilic infiltration of the mucosal layers, suggesting a diagnosis of allergic proctocolitis; in others, signs compatible with abnormal circulatory regulation [61]. Moak et al., studying preterm neonates, observed similarities between NEC and CMPA. They developed three theories to explain a possible overlap between CMPA and NEC: CMPA is an entity that is misdiagnosed as NEC; CMPA is a pre-existing condition that increases the risk of NEC; and NEC can lead to subsequent cow’s milk allergy [62].

Finally, D’Auria et al. [27] conducted a retrospective single-center study in a neonatal intensive care unit. All preterm newborns with isolated rectal bleeding (N = 43) were included. At symptom onset, all infants were kept fasting for 48 h and underwent diagnostic evaluation, including blood tests, abdominal radiographs, abdominal ultrasound, and stool infection tests. The NEC diagnosis was based on modified Bell’s criteria [63]. Patients who had NEC excluded were fed an amino acid-based formula, and after one week, an oral food challenge was performed to confirm or rule out CMPA. Infants whose symptoms recurred (e.g., presence of blood in stools after reintroduction of cow’s milk formula) were diagnosed with CMPA and continued an amino acid formula, whereas those without recurrence after reintroduction of cow’s milk formula were diagnosed with idiopathic transient neonatal colitis. Eighteen patients were diagnosed with NEC, 16 with CMPA, and 9 with transient colitis [27]. This seems to be a reasonable approach to the management of these neonates.

This review suggests that necrotizing enterocolitis should always be considered in the differential diagnosis of IRB and that, after NEC has been ruled out, the diagnosis of CMPA should be confirmed precociously by an oral food challenge in clinically stable neonates with rectal bleeding. Other diagnoses must be investigated with clinical, laboratory, or imaging when deemed necessary. Examination of the mother’s nipples, the anal region of newborns, and microbiological studies — especially if diarrhea is present. Under appropriate medical supervision, this approach can help identify the subgroup of infants who might benefit from a cow’s milk-free diet.

## Authors’ contributions

Conceptualization: Alexandra Markevich, Maria Ângela Bellomo Brandão, Elizete A. Lomazi

Project administration: Alexandra Markevich, Elizete A. Lomazi

Recruitment and data acquisition: Alexandra Markevich, Júlia G. Botelho, Luana L. Morikawa, Maria Ângela Bellomo Brandão, Elizete A. Lomazi

Analysis and interpretation of data: Alexandra Markevich, Júlia G. Botelho, Luana L. Morikawa, Maria Ângela Bellomo Brandão, Elizete A. Lomazi

Manuscript writing: Alexandra Markevich, Júlia G. Botelho, Luana L. Morikawa, Maria Ângela Bellomo Brandão, Elizete A. Lomazi

Manuscript revision and technical support: Alexandra Markevich, Júlia G. Botelho, Luana L. Morikawa, Maria Ângela Bellomo Brandão, Elizete A. Lomazi

## Conflicts of interest

The authors declare no conflicts of interest.

## Data Availability

The data that support the findings of this study are available in the Open Science Framework platform.

## Appendix Supplementary materials

Image, application 1

## Footnotes

Supplementary material associated with this article can be found in the online version at doi:10.1016/j.jped.2026.101594.
