# Supplementary material for: Isolated rectal bleeding in neonates: a scoping review
Source: J Pediatr (Rio J). 2026 Aug 12;102(5):101594. doi: 10.1016/j.jped.2026.101594 (PMC13486324; doi:10.1016/j.jped.2026.101594)
Supplement: Supplementary file 1 [file mmc1.docx]

**JPED-D-26-00115**

**Supplementary material**

**Appendix I – Search Strategies**

| Database | Subject Headings | Descriptors and free terms used in the search strategy | | | | |
| --- | --- | --- | --- | --- | --- | --- |
|  |  | 1 | 2 | 3 | 4 | 5 Free Term – Synonyms in EMTREE |
| PUBMED | MeSH Medical Subject Headings | "Infant, Newborn" | Hemorrhage | "Gastrointestinal Hemorrhage" | Rectum | "Rectal bleeding" |
| PUBMED PMC | MeSH - Medical Subject Headings | "Infant, Newborn" | Hemorrhage | "Gastrointestinal Hemorrhage" | Rectum | "Rectal bleeding" |
| BVS / BIREME | DeCS | "Infant, Newborn" | Hemorrhage | "Gastrointestinal Hemorrhage" | Rectum | "Rectal bleeding" |
| EBSCOHOST |  | "Infant, Newborn" | Hemorrhage | "Gastrointestinal Hemorrhage" | Rectum | "Rectal bleeding" |
| Scopus |  | "Infant, Newborn" | Hemorrhage | "Gastrointestinal Hemorrhage" | Rectum | "Rectal bleeding" |
| WEB OF SCIENCE |  | "Infant, Newborn" | Hemorrhage | "Gastrointestinal Hemorrhage" | Rectum | "Rectal bleeding" |
| EMBASE | Emtree | "Infant, Newborn" | Hemorrhage use preferred term: bleeding | Gastrointestinal Hemorrhage | Rectum | Rectal bleeding  use preferred term: rectum hemorrhage |
| Cochrane Library | MeSH - Medical Subject Headings | "Infant, Newborn" | Hemorrhage | "Gastrointestinal Hemorrhage" | Rectum | "Rectal bleeding" |
| PROQUEST | MeSH - Medical Subject Headings | "Infant, Newborn" | Hemorrhage | "Gastrointestinal Hemorrhage" | Rectum | "Rectal bleeding" |

| Database | Subject Headings | Descriptors and free terms used in the search strategy | |
| --- | --- | --- | --- |
|  |  | Free Term – Synonyms in EMTREE |  |
| PUBMED | MeSH - Medical Subject Headings | "rectum haemorrhage" OR "rectum bleeding" OR proctorrhage OR proctorrhagia OR proctorrhagy OR "rectal haemorrhage" OR "rectal hemorrhage" OR "rectocolic bleeding" OR "haemorrhage, rectum" OR "hemorrhage, rectum" | Proctocolitis |
| PUBMED PMC | MeSH - Medical Subject Headings | "rectum haemorrhage" OR "rectum bleeding" OR proctorrhage OR proctorrhagia OR proctorrhagy OR "rectal haemorrhage" OR "rectal hemorrhage" OR "rectocolic bleeding" OR "haemorrhage, rectum" OR "hemorrhage, rectum" | Proctocolitis |
| BVS / BIREME | DeCS | "rectum haemorrhage" OR "rectum bleeding" OR proctorrhage OR proctorrhagia OR proctorrhagy OR "rectal haemorrhage" OR "rectal hemorrhage" OR "rectocolic bleeding" OR "haemorrhage, rectum" OR "hemorrhage, rectum" | Proctocolitis |
| EBSCOHOST |  | "rectum haemorrhage" OR "rectum bleeding" OR proctorrhage OR proctorrhagia OR proctorrhagy OR "rectal haemorrhage" OR "rectal hemorrhage" OR "rectocolic bleeding" OR "haemorrhage, rectum" OR "hemorrhage, rectum" | Proctocolitis |
| Scopus |  | "rectum haemorrhage" OR "rectum bleeding" OR proctorrhage OR proctorrhagia OR proctorrhagy OR "rectal haemorrhage" OR "rectal hemorrhage" OR "rectocolic bleeding" OR "haemorrhage, rectum" OR "hemorrhage, rectum" | Proctocolitis |
| WEB OF SCIENCE |  | "rectum haemorrhage" OR "rectum bleeding" OR proctorrhage OR proctorrhagia OR proctorrhagy OR "rectal haemorrhage" OR "rectal hemorrhage" OR "rectocolic bleeding" OR "haemorrhage, rectum" OR "hemorrhage, rectum" | Proctocolitis |
| EMBASE | Emtree | "rectum haemorrhage" OR "rectum bleeding" OR proctorrhage OR proctorrhagia OR proctorrhagy OR "rectal haemorrhage" OR "rectal hemorrhage" OR "rectocolic bleeding" OR "haemorrhage, rectum" OR "hemorrhage, rectum" | Proctocolitis |
| Cochrane Library | MeSH - Medical Subject Headings | "rectum haemorrhage" OR "rectum bleeding" OR proctorrhage OR proctorrhagia OR proctorrhagy OR "rectal haemorrhage" OR "rectal hemorrhage" OR "rectocolic bleeding" OR "haemorrhage, rectum" OR "hemorrhage, rectum" | Proctocolitis |
| PROQUEST | MeSH – Medical Subject Headings | "rectum haemorrhage" OR "rectum bleeding" OR proctorrhage OR proctorrhagia OR proctorrhagy OR "rectal haemorrhage" OR "rectal hemorrhage" OR "rectocolic bleeding" OR "haemorrhage, rectum" OR "hemorrhage, rectum" | Proctocolitis |

**Appendix II – Characteristics of included studies**

**Case-control**

| **Author (Year)** | **Country** | **N (cases)** | **Etiologies** |
| --- | --- | --- | --- |
| Maayan-Metzger et al (2010) (5)  Said et al (2014) (6)  Rallis et al (2021) (7) | Israel  France  Greece | 147  33  71 | 147 IRB 33 IRB 46 hemorrhagic colitis; 25 CMPA |

IRB: Isolated rectal bleeding. CMPA: Cow’s milk protein allergy.

**Case series**

| **Author (Year)** | **Country** | **N (cases)** | **Etiologies** |
| --- | --- | --- | --- |
| Levene et al (1979) (8)  Lake et al (1982) (9)  Dupont et al (1987) (10)  Taxman et al (1987) (11)  Teach et al (1994) (12)  De la Torre Mondragón et al (1995) (13)  Canioni et al (1997) (14)  Haghighat et al (2003) (15)  Sorea et al (2003) (17)  Maayan-Metzger et al (2004) (1)  Sierra Salinas et al (2006) (18)  Ohtsuka et al (2007) (19)  Lucarelli et al (2011) (21)  Zahmatkeshan et al (2012)(16)  Jang et al (2012) (22)  Mori et al (2014) (20)  Gupta et al (2016) (23)  Miyazawa et al (2016) (24)  Ahmadipour et al (2020) (25)  Lin et al (2022) (26)  D’Auria et al (2024) (27) | England  USA  France  USA  USA  Mexico  France  Iran  France  Israel  Spain  Japan  Italy  Iran  South Korea  Japan  Canada  Japan  Iran  China  Italy | 50  6  8  41  13  1  17  1  2  307  4  2  5  1  13  21  6  11  60  269  25 | 44 IRB; 6 anal fissure 6 CMPA 7 IRB; 1 CMPA 17 IRB; 24 allergic colitis 9 CMPA; 2 anal fissure; 1 vaginal bleeding; 1 IRB 1 Colonic angiodysplasia 17 Ecchymotic colitis 1 Nodular Lymphoid Hyperplasia 2 CMPA 307 IRB 4 CMPA 2 Nodular Lymphoid Hyperplasia 5 CMPA 1 Nodular Lymphoid Hyperplasia 12 Neonatal transient colitis; 1 CMPA 9 Neonatal transient colitis. 12 CMPA 2 Viral infection; 1 anal fissure; 3 CMPA 9 IRB; 2 CMPA 60 CMPA 153 CMPA; 50 swallowed maternal blood; 31 viral infection; 22 IRB; 13 anal fissure 9 Neonatal transient colitis; 16 IRB |

IRB: Isolated rectal bleeding. CMPA: Cow’s milk protein allergy.

**Case reports**

| **Author (Year)** | **Country** | **N (cases)** | **Etiologies** |
| --- | --- | --- | --- |
| Youngs ER et al (1985) (31)  Perisic VN et al (1988) (32)  Wilson NW et al (1990) (33)  Feiterna-Sperling C et al (1997) (34)  Strauss RS et al (1998) (35)  Kumar D et al (2000) (36)  Hatzidaki EG et al (2003) (37)  Swart JF et al (2003) (38)  Faber MR et al (2005) (39)  Tien CH et al (2006) (30)  Piñera MJ et al (2007) (40)  Al-Mehaidib A et al (2009) (41)  Dargent JL et al (2009) (42)  Shankarnaryanan S et al (2012) (43)  Alabsi HS et al (2013) (44)  O'Neil AM et al (2016) (45)  Joshi A et al (2016) (46)  Debuf MJ et al (2017) (47)  Hirschel J et al (2018) (48)  Aissa K et al (2019) (49)  Thus KA et al (2019) (50)  Iannotta et al (2020) (51)  Abusalah ZG et al (2020) (52)  Zárubová P et al (2020) (53)  Lu Y et al (2021) (29)  Guanà et al (2021) (54)  Nii K et al (2021) (55)  Shehab N et al (2021) (56)  Hoshi K et al (2023) (57)  Barea Domínguez M et al (2024) (58)  Ruesen J et al (2025) (28)  Servitje-Verdaguer B et al (2025) (59) | England  Yugoslavia  USA  Germany  USA  USA  Greece  Netherlands  Netherlands  China  Spain  Saudi Arabia  Belgium  Australia  USA  USA  Nepal  Belgium  Switzerland  France  Netherlands  Italy  United Arab Emirates  Czech Republic  China  Italy  Japan  USA  Japan  Spain  Switzerland  Spain | 3  1  1  1  1  3  1  1  1  1  1  1  1  1  1  1  1  1  1  1  1  1  4  2  1  1  1  1  1  1  1  1 | *Campylobacter* infection CMPA CMPA CMPA CMPA Allergic colitis CMPA CMPA CMPA CMPA Colonic duplication and Meckel’s diverticulum Angiodisplasia Allergic colitis Intramural hematoma Eosinophilic gastroenteritis CMPA Gastric ulcer Transient eosinophilic colitis *Campylobacter* infection CMPA *Campylobacter* infection Medication (sildenafil) Amebiasis *Campylobacter* infection FPIES Meckel’s diverticulum Maternal medication (Mesalazine) Swallowed maternal blood Transient eosinophilic colitis Transient eosinophilic colitis CMPA Transient eosinophilic colitis |

CMPA: Cow’s milk protein allergy. FPIES: *Food protein induced proctocolitis*.
